# Dengue-2 Structural Proteins Associate with Human Proteins to Produce a Coagulation and Innate Immune Response Biased Interactome

**DOI:** 10.1186/1471-2334-11-34

**Published:** 2011-01-31

**Authors:** Brenda B Folly, Almeriane M Weffort-Santos, CG Fathman, Luis RB Soares

**Affiliations:** 1 Federal University of Paraná, Pharmaceutical Sciences Post-graduation Program, Av. Pref. Lothário Meissner 632, CEP 80210-170, Curitiba-PR, Brazil; 2 Federal University of Paraná, Medical Pathology Department, Av. Pref. Lothario Meissner 632, CEP 80210-170, Curitiba-PR, Brazil; 3 Stanford University School of Medicine, Department of Medicine, Division of Immunology & Rheumatology, CCSR Building, 269 Campus Drive, Room 2225 Stanford, CA 94305-5166, USA; 4 Instituto Pelé Pequeno Príncipe, Hospital Pequeno Príncipe, Av. Silva Jardim, 1632 - Curitiba - PR - CEP 80250-060, Brazil

## Abstract

**Background:**

Dengue virus infection is a public health threat to hundreds of millions of individuals in the tropical regions of the globe. Although Dengue infection usually manifests itself in its mildest, though often debilitating clinical form, dengue fever, life-threatening complications commonly arise in the form of hemorrhagic shock and encephalitis. The etiological basis for the virus-induced pathology in general, and the different clinical manifestations in particular, are not well understood. We reasoned that a detailed knowledge of the global biological processes affected by virus entry into a cell might help shed new light on this long-standing problem.

**Methods:**

A bacterial two-hybrid screen using DENV2 structural proteins as bait was performed, and the results were used to feed a manually curated, global dengue-human protein interaction network. Gene ontology and pathway enrichment, along with network topology and microarray meta-analysis, were used to generate hypothesis regarding dengue disease biology.

**Results:**

Combining bioinformatic tools with two-hybrid technology, we screened human cDNA libraries to catalogue proteins physically interacting with the DENV2 virus structural proteins, Env, cap and PrM. We identified 31 interacting human proteins representing distinct biological processes that are closely related to the major clinical diagnostic feature of dengue infection: haemostatic imbalance. In addition, we found dengue-binding human proteins involved with additional key aspects, previously described as fundamental for virus entry into cells and the innate immune response to infection. Construction of a DENV2-human global protein interaction network revealed interesting biological properties suggested by simple network topology analysis.

**Conclusions:**

Our experimental strategy revealed that dengue structural proteins interact with human protein targets involved in the maintenance of blood coagulation and innate anti-viral response processes, and predicts that the interaction of dengue proteins with a proposed human protein interaction network produces a modified biological outcome that may be behind the hallmark pathologies of dengue infection.

## Background

Dengue virus infections affects scores of people world-wide and represent a serious, recurrent public health and social-economical problem, especially in developing countries. Although the disease usually manifests itself in its mildest form, dengue fever, severe forms of the disease: dengue hemorrhagic fever and dengue shock syndrome frequently arise, and are responsible for the majority of dengue related deaths, especially in children. The pathophysiological mechanisms that distinguish between the disease forms are still not well understood, but among all variables, levels of viremia seem to correlate best with disease outcome. Current models of virus assembly and export indicate that three types of dengue virus particles co-exist during the viral infection cycle: (*i*) mature particles containing the structural proteins E, cap and M, (*ii*) immature particles containing the structural proteins E, Cap and PrM and (*iii*) a third kind of particle, representing partially mature virions, often found in the supernatants following replication of DENV-2 virus in cultured insect cells. These retain the full unprocessed prM protein and may represent up to 40% of all extracellular particles in that setting. We reasoned that a more detailed knowledge of the protein interaction partners of these proteins might provide important clues to help understand the biology of the host-dengue virus relationship, and possibly help to uncover novel avenues for therapeutic intervention. Our data, from two-hybrid technology and systems biology tools, provide evidence that dengue virus structural proteins establish direct interactions with human proteins participating in crucial coagulation and inflammatory responses. These observations may help to explain the faulty behavior of the coagulation pathway in subjects infected by dengue virus.

## Methods

### Media and chemicals

Luria-Bertani (LB) liquid media, LB-agar and common molecular biology reagents were purchased from Invitrogen (Invitrogen, Carlsbad, CA). Antibiotics and X-gal used in the two-hybrid screen were from Sigma (Sigma Aldrich, St. Louis, MO), and were prepared as fresh stock solutions prior to each assay. Oligonucleotide primers used for the amplification of the cDNA for dengue structural proteins were obtained from the Stanford University PAN facility. PCR amplification was performed with Ultra-PFU (Agilent Technologies, Santa Clara, CA) according to the manufacturer's instructions.

### Bacterial two-hybrid screens

A dengue-2 virus cDNA derived from the dengue-2 infectious clone 16681 (a kind gift from Mitchell Lunn and Karla Kirkegaard, Stanford University) was used as a template for PCR amplification of the Env, PrM and Cap coding sequences according to the published sequence [1]. cDNAs were originally cloned into the pCR4-TOPO blunt vector (Invitrogen, Carlsbad, CA), fully sequenced and then subcloned into the bait vector pBT (Agilent Technologies, Santa Clara, CA), and again sequenced to verify the open reading frame continuity with the fusion partner. pTRG plasmids harboring human liver and whole brain cDNA libraries were obtained from Agilent, and handled according to the manufacturer's instructions. Bacterial two hybrid screens were performed according to the manufacturer's manual, with some modifications [2] in order to decrease the rate of false positives. Briefly, after transformation of the amp_LacZ reporter cells, carbenicilin-resistant (250 ug/ml) positive colonies were replated at increasing carbenicillin concentrations, and colonies still scoring positives with a concentration of 350 ug/ml or higher were used for the secondary screen, with LacZ. At this stage, only the colonies with an intense blue coloration were selected for revalidation and sequencing. In a previous work (our unpublished observations), these modifications resulted in validation rates above 75% when putative interaction partners were assayed by co-immunoprecipitation in mammalian cells.

### Construction and analysis of the interactome networks

All network graphical assembly and manipulations were performed on Cytoscape [3]. For the construction of the human protein interaction network, physical binary interactions were imported from the web through the Pathway Commons Cytoscape plug-in [4], and further enriched with information retrieved directly from the BioGrid [5], HPRD [6], DIP [7] and IntAct [8] databases. The NCBI gene name attribute was used to unify the protein lists and is used throughout this paper. The dengue-human primary network was built manually in Cytoscape, and attributes for each gene given name (NCBI gene name) were imported through the Biomart plug-in [9]. The network for the dengue primary interactors direct neighbors (level 2) was built from within the human network by creating a group (through the group tool plug-in) consisting of DENV2-human interacting proteins. The group was selected and served to populate a sub network with the first neighbors and adjacent edges into a unified interactome. Topological analysis of individual and combined networks was performed with Network Analyzer, a Cytoscape plug-in that allows analysis and visualization of network topological features [10].

### Functional analysis

Gene ontology enrichment and pathway analysis was performed with either BinGO [11], DAVID [12,13], Webgestalt [14] or directly at KEGG [15,16]. BinGO, DAVID and Webgestalt built-in statistical modules automatically compute the enrichment of specific pathways or gene ontology terms for every binary interaction in the network, and can be customized to calculate significance by the Fisher's exact test and the multiple test correction techniques Benjamini, Bonferroni and FDR for larger gene lists. Pathways retrieved from KEGG were compared to similar pathway denominations at the Reactome, Wiki Pathways and the InnateDB databases for additional components and accuracy.

### Microarray analysis

Datasets of dengue infection experiments deposited either at the GEO database http://www.ncbi.nlm.nih.gov/geo/ or ArrayExpress http://www.ebi.ac.uk/microarray-as/ae/ were imported as raw signal (CEL files) values into Genespring GX11 using built in import modules for Affymetrix arrays. Values were normalized by log transformation and group replicates compared by either unpaired t-test or two-way ANOVA, depending on the dataset. Fold-change analysis was used to compare expression between groups.

## Results and Discussion

**Figure 1:**
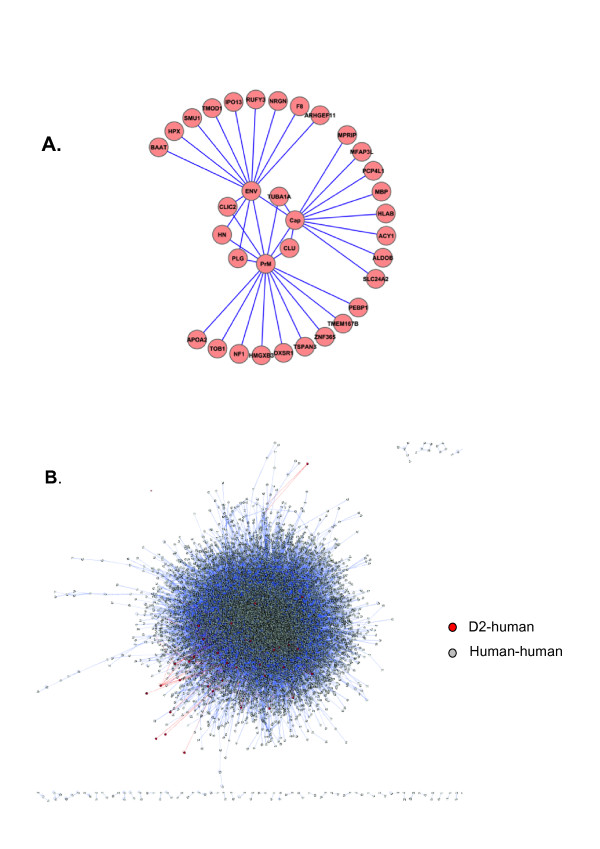
**Graphical representation of the DENV2 structural protein interactome**. **(A) **Primary network formed by the D2 structural proteins and its targets, obtained experimentally in the two-hybrid assay. **(B) **Topological representation of the network in (A) overlayed on a human-human interaction network. Red nodes and red edges represent the nodes and edges seen in "A" and blue nodes and edges the remainder of the human-human network.

To study dengue-human protein-protein interactions, we individually cloned the Env, PrM and Cap genes from a DENV2 [1] isolate into the bacterial two-hybrid vector pBT. Interactions were identified in the bacterial two-hybrid screen by assaying the individual baits against a whole brain, and a liver cDNA library, both cloned into pTRG vectors. In total, 10^6 ^clones were screened in each library. Carbenicillin resistant beta-Gal positive colonies were retested at higher stringency conditions by gradually increasing the dose of carbenicillin. Surviving colonies were re-assayed for beta-Gal activity, and colonies staining for the highest intensity beta-Gal expression (blue coloration) were selected and sequenced. Forty-seven, and 30 in-frame sequences were obtained for the brain and liver screens, respectively, representing 31 unique proteins, presented here in Cytoscape (**Figure **1A and **Table **1). A few human proteins in our screen interacted with more than one viral protein, a feature also found in a recent HCV interactome study, and attributed to a biological "essentiality" of these host proteins for the virus life cycle.

**Table 1:** Gene ID, Gene Symbol and common name for the proteins rescued from the two-hybrid assay

BAIT	SYMBOL	GENE ID	NAME
Cap	ACY1	95	Aminoacylase 1

Cap	ALDOB	229	Aldolase B, fructose-bisphosphate

PrM	APOA2	336	Apolipoprotein A-II

Env	ARHGEF11	9826	Rho guanine nucleotide exchange factor (GEF) 11

Env	BAAT	570	Bile acid Coenzyme A: amino acid N-acyltransferase (glycine N-choloyltransferase)

Env/PrM	CLIC2	1193	Chloride intracellular channel 2

Cap/PrM	CLU	1191	Clusterin

Env	F8	2157	Coagulation factor VIII, procoagulant component

Cap	HLAB	3106	Major histocompatibility complex, class I, B

PrM	HMGXB3	22993	HMG box domain containing 3

Env/PrM	HN	*Q8IVG9	Humanin (*UniProt ID)

Env	HPX	3263	Hemopexin

Env	IPO13	9670	Importin 13

Cap	MBP	4155	Myelin basic protein

Cap	MFAP3L	9848	Microfibrillar-associated protein 3-like

Cap	MPRIP	23164	Myosin phosphatase Rho interacting protein

PrM	NF1	4763	Neurofibromin 1

Env	NRGN	4900	Neurogranin (protein kinase C substrate, RC3)

PrM	OXSR1	9943	Oxidative-stress responsive 1

Cap	PCP4L1	654790	Purkinje cell protein 4 like 1

PrM	PEBP1	5037	Phosphatidylethanolamine binding protein 1

Env/PrM	PLG	5340	Plasminogen

Env	RUFY3	22902	RUN and FYVE domain containing 3

Cap	SLC24A2	25769	Solute carrier family 24 (sodium/potassium/calcium exchanger), member 2

Env	SMU1	55234	Smu-1 suppressor of mec-8 and unc-52 homolog (C. elegans)

PrM	TMEM167B	56900	Transmembrane protein 167B

Env	TMOD1	7111	Tropomodulin 1

PrM	TOB1	10140	Transducer of ERBB2, 1

PrM	TSPAN3	10099	Tetraspanin 3

Cap/PrM	TUBA1A	7846	Tubulin, alpha 1a

PrM	ZNF365	22891	Zinc finger protein 365

Notice that for the gene coding for the functional peptide Humanin, the UniProt ID is given.

From the 31 interactions catalogued, 4 appear to be essential nodes in the human protein interaction network, as knockout of genes encoding these proteins in mice can be either embryonic lethal (NF1, OXSR1 and TMOD1) or shorten life span considerably (MBP). Another 11 interactions take place with proteins whose mutations cause disease in man or mice (APOA2, TOB1, PEBP1, SLC24A2, F8, HPX, NRGN, HPX, CLU, PLG and TUBA1A) [17,18] indicating the importance of these proteins in maintaining network structure, that is, the cell's biological functions. While the set of 31 identified proteins is in itself, however, poorly connected, as there are only 2 inter-connecting edges, collectively, these proteins are critical for host development and/or survival.

An ontological analysis of the putative interactors using DAVID [12,13] revealed that the gene ontology groups "Response to stress", "Wound healing" and "Protein import" were overrepresented in the dataset (**Table **2). In addition, proteins from the dataset for the KEGG pathways shows overrepresentation for a single pathway, "Complement and coagulation cascades" and corresponds to the Plasminogen (PLG) and Factor VIII (F8) proteins (**Table **2). Binding to and altering the properties for plasminogen and factor VIII may be the molecular basis to link the clinical and pathological relationship between dengue infection and haemostatic abnormalities such as vascular leakage, thrombocytopenia and hemorrhage [19,20]. Several reports have shown that cell surface receptor-bound plasminogen, or plasminogen coupled to its soluble receptor, are more readily activated to plasmin than free plasminogen [21-23]. Thus, it is possible that at high viremia, the availability of free plasminogen for cell surface binding is decreased, resulting in down-regulation of cell-bound dependent plasmin activation of matrix-degrading proteinases [24]. If the association with factor VIII proves to be inhibitory, then a combined effect of increased plasmin conversion and a factor VIII "deficiency" may represent a negative double hit, and could be at least be partially responsible for the haemostatic imbalance seen in dengue infections (**supplemental figure S1**, see **Additional file **1). Recently it became clear that dengue viral particles have intimate interactions with platelets, not only associated at the cell surface, but also using this cell type as transient replication hosts. This a critical point, as platelet surface assembly of tenase (factor VIII/IX) and plasminogen conversion may become deregulated upon viral binding. Adding to this scenario it has been proposed that the degree of haemostatic imbalance in dengue fever is also highly influenced by the complement system [25]. As reported before for the non-structural dengue protein NS1 [26], we show here that DENV2 PrM also binds to clusterin (CLU) in our two-hybrid assay. The authors in the previous report [26] hypothesize that the association of NS1 with clusterin may free C7, which normally associates with clusterin, therefore facilitating the formation of the terminal complement complex (TCC). It is possible that the association with PrM also produces a similar scenario and, in addition, facilitates immune evasion by inhibiting the formation of the membrane attack complex (MAC) at the virion surface which is commonly decorated with anti-E/PrM antibodies. This is reportedly a common solution adopted by a diverse group of enveloped viruses as HIV, HBV and Poxviruses, which either incorporate host-derived anti-complement factors in their envelope, e.g. CD59, or, for large genome viruses, encode complement regulator genes [27]. Thus, although it is understandable that viruses exploit the complement pathway for avoiding lysis and at the same time enhance virus uptake, the reason for their involvement with the coagulation pathway is not clear. It is possible that this is just a facet of another intrinsic immune defense hurdle that the virus has to overcome, given the fact that deposition and polymerization of fibrin on the surface of microorganisms (albeit not yet demonstrated for dengue virus particles) has been shown, and is proposed to be, one way the host may impose barriers on the infecting agent to curtail dissemination of infection [28,29]. Another intriguing possibility is that the binding of the structural PrM and Env to PLG and CLU strengthen the already existing connectivity between the complement pathway and fibrinolytic activity at the interactome level [30,31], via CLU-C7-PLG and CLU-PRNP-PLG, causing "oversensitivity" in the system.

**Table 2:** Gene ontology categories and pathways over-represented for the proteins shown in the network in Figure 1A, according to DAVID

GENE ONTOLOGY	p-VALUE	GENES
Response to stress	1.50E-03	CLU, OXSR1, PLG, F8, ALDOB, TMOD1, NF1

Wound healing	1.10E-02	PLG, F8, NF1

Protein import	8.00E-03	IPO13, TOB1, NF1

PATHWAYS - KEGG	p-VALUE	GENES

Complement and coagulation cascades	9.40E-02	PLG, F8

Gene ontology (Biological Process) and Pathway (KEGG) terms, p-value (calculated against DAVID's human reference set) and gene names are given.

To gain a better insight into the working of the protein interaction network during dengue infection, we manually increased our primary network by using data from the Pathway Commons database Cytoscape import plug-in (4) to include proteins interacting with the primary dengue structural protein interactors (immediate neighbors), and overlaid this on a manually curated human PPI made of the union of networks retrieved from four databases, HPRD [6], Biogrid [5], DIP [7] and IntAct [8]. The condensed human PPI has 11.479 nodes and 52.208 edges to which our primary 31 interactors made 351 connections (**Figure **1B). A topological analysis of this DENV2-human (D2-H) protein interaction network revealed that the average degree of the primary 31 interactors is 11.19 (+/- 12.5, range 2-52) as 27 of the 31 primary targets were represented in the condensed human PPI network. This is an increase of over 75% in connectivity compared to the average degree of our human PPI network, which is 6.93, and indicates that the dengue structural proteins are highly connected in the human interactome. Although our D2-H interactome is only partial, not accounting for the non-structural proteins, the high connectivity observed is in line with results from other viral interactome studies such as the EBV-human interactome (average degree 15) and the HCV-human interactome (average degree 15.6). Other topological features of the D2-human interaction network are an average between the value of 2.1 × 10^-4 ^(compared to 1.4 × 10^-4 ^for our human-human reference interactome) and an average shortest path of 3.1 (compared to 3.8 for the reference network). Together the result of these studies suggest, as was proposed before [32,33], that high connectivity may indeed be a common feature of viral-human interactomes and would be born out of the necessity of the virus to hijack major cellular systems in order to replicate the viral genome and produce virions. Given the fact that in biological networks, nodes with a number of interactions above the average, called hubs, are usually essential, as its loss results in severe phenotypes [34], it appears that the infection network constructed for HCV, EBV and the DENV2 infection network shown here, corroborate that assertion.

**Figure 2:**
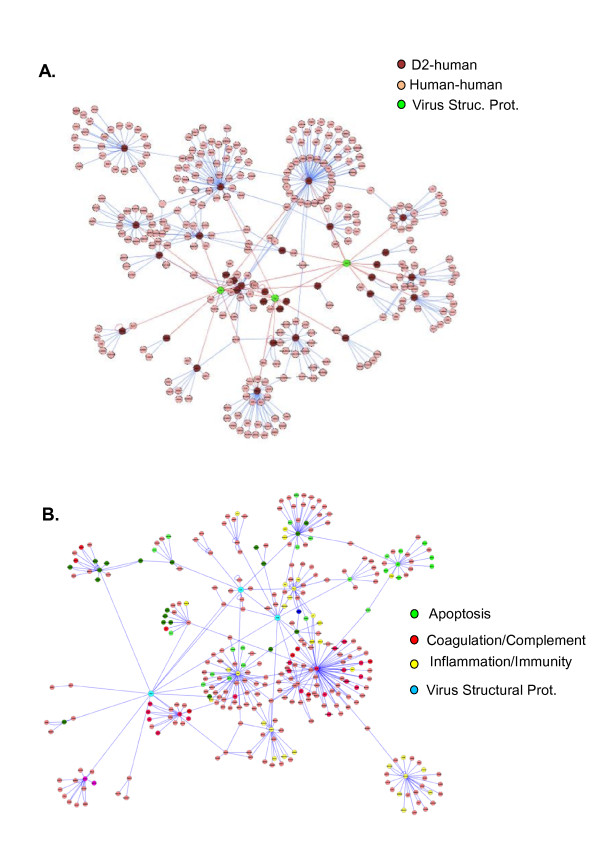
**(A) Graphical representation of the interaction network from "figure 1A" after enrichment with first neighbors (level 2 interactions) for the 31 primary interactors**. In **(B)**, the same network seen in "A" with the nodes colored according to their gene ontology classifications.

Ontological analysis of the D2-H network, now including the level 2.351 interactions, again making use of DAVID, revealed that the categories "Blood coagulation", "Regulation of body fluid levels", "Cell death", "Response to wounding" and "Acute inflammatory response" were overrepresented (**Table **3**and Figure **2B) as were the KEGG pathways "Complement and coagulation cascades", "MAPK signaling", "TGF-beta signaling", "Focal adhesion", "Adherens junctions" and "Toll-like receptor signaling" (**Table **3). Particularly important to this analysis, it seems that the dengue virus targets distinct pathways, as it has to deal with distinct biological processes in order to achieve stable infection.

**Table 3:** Gene ontology categories and pathways over-represented for the proteins shown in the network in Figure 1C and 1D (level 2 interactions), according to DAVID

GENE ONTOLOGY	*p-VALUE*	GENES
Blood coagulation	2.50E-18	CXCL2, STK39, SERPING1, MAPK9, LMAN1, MAPK1, HSPA5, F10, MAPK11, PLG, MDK, HMGB1, EGFR, PRKCG, PLAT, THBS1, F3, MAPK13, F2R, PRNP, BCL2, HSP90AA1, ALDOB, SERPINF2, VWF, OXSR1, MAP2K1, ALB, C7, RPS6KA5, MAPKAPK5, PRKCA, F8, PROC, KNG1, NF1, TFPI2, GRM7, CLU, SERPINE1, SFN, PLA2G7, TLK2, MAPK14, C8B, XRCC6, GNA12, SERPINC1, APTX, MMP25, C9, TRIB3, F2, TMOD1, FN1, F2RL1, PLAU, BMPR1B, TLK1, CEBPB, APOH, HSPA8, COL1A, BAX, PROS1, TF, VCP, F9, GNA13

Regulation of body fluid levels	3.30E-16	SERPING1, LMAN1, F2R, F8, PROC, KNG1, F2, F10, TFPI2, F2RL1, VWF, SERPINE1, PLG, PLAU, APOH, PLAT, PROS1, GNA12, THBS1, F3, F9, SERPINC1, GNA13

Cell death	4.90E-13	PRKCZ, MAPK1, HSPA, NEB, CALR, PLG, HMGB1, MARK4, PRKCG, MAP3K7, PPARD, F2R, PRNP, BCL2, APP, RAF1, C7, ALB, IGFBP3, BCL2L1, PRKCA, ATN1, PROC, KNG1, NF1, PAK1, CLU, SFN, MCL1, BAK1, SIAH1, C8B, SNCA, BCL2L10, APTX, TRAF6, C9, TUBB, F2, TRIB3, STK4, PAX3, SMAD3, CEBPB, RTN4, BAX, ATXN1, VCP, MMP9

Response to wounding	2.70E-16	CXCL2, SERPING1, LMAN1, PRKCA, F8, KNG1, PROC, F10, NF1, TFPI2, SERPINE1, CLU, PLG, MDK, PLA2G7, C8B, PLAT, F3, THBS1, GNA12, SERPINC1, F2R, MMP25, C9, F2, SERPINF2, FN1, F2RL1, VWF, PLAU, BMPR1B, CEBPB, APOH, C7, BAX, TF, PROS1, F9, GNA13

Acute inflammatory response	8.50E-07	SERPING1, CLU, F8, C9, F2, CEBPB, C7, TF, C8B, SERPINF2, FN1

PATHWAYS	p-VALUE	GENES

Complement and coagulation cascades	8.90E-14	SERPING1, F2R, C9, F8, PROC, KNG1, F2, F10, SERPINF2, VWF, SERPINE1, PLG, PLAU, C7, CPB2, PROS1, PLAT, C8B, F3, F9, SERPINC1

MAPK signaling pathway	1.90E-08	MAPKAPK5, MAPK9, PRKCA, MAPK1, NF1, MKNK1, PAK1, MAPK11, PRKCB1, CHUK, EGFR, MAPK14, PRKCG, MAP3K7, GNA12, MYC, TRAF6, MAPK13, RPS6KA1, STK4, TGFBR2, MAP3K14, MAP2K1, NGFB, IKBKB, RAF1, RPS6KA5, TGFBR1, PRKACA, MAPK3

TGF-beta signaling pathway	4.60E-07	SMAD2, SMAD9, SMURF1, SMAD6, MAPK1, TGFBR2, RHOA, BMPR1B, SMAD3, SMAD1, TGFBR1, MAPK3, THBS1, SMAD5, SMAD4, MYC

Focal adhesion	2.10E-05	LAMA1, LAMA5, MAPK9, PRKCA, MAPK1, BCL2, SRC, FN1, PAK1, VWF, RHOA, ERBB2, PRKCB1, MAP2K1, RAF1, EGFR, COL1A1, PRKCG, THBS1, MAPK3, LAMA3

Adherens junction	5.20E-05	SMAD2, TGFBR2, ERBB2, RHOA, MAPK1, SRC, SMAD3, EGFR, TGFBR1, MAP3K7, MAPK3, SMAD4

Toll-like receptor signaling pathway	9.30E-04	MAPK9, MAPK11, MAPK3, MAPK13, TRAF6, MAPK1, CHUK, MAP2K1, IKBKB, MAPK14, IRAK1, AP3K7, MAPK3

Gene ontology terms (Biological Process) and Pathway (KEGG) terms, p-value (calculated against DAVID's human reference set) and gene names are given.

The association of the Env protein with the cytoskeletal module via ARHGEF11 and RhoA may play an important role during viral entry, as reorganization of the cortical actin cytoskeleton into filopodia is essential for DENV2 infection of HMEC-1 cells [35]. ARHGEF11 (PDZ-RHOGEF) is a guanine exchange factor for Rho proteins and is an upstream regulator of Rho-dependent actin reorganization downstream from diverse receptors. Interestingly, DC-SIGN, a putative dengue virus receptor uses LARG (ARHGEF12), an ARHGEF11 close homologue, to activate Rho in association with HIV infection [36]. In addition MRIP, another regulator of Rho-dependent actin rearrangement [37], associates directly with the DENV-2 Capsid (Cap) protein. Together with the data showing that Env interacts with tropomodulin1 (TMOD1) (**Figure **2A) it allow us to speculate that the interactions of dengue structural proteins with members of the actin remodeling machinery may act in an inhibitory fashion as both Rho activation and TMOD1 inhibition of elongation favor the formation of stress fibers, rigid structures which unlike filopodia may interfere negatively with virus entry. Another Env interaction we detected may be involved with F-actin dynamics and filopodia formation. RUFY3 (also known as Singar1 or RIPX) is a putative inhibitor of PI3K and is predicted to have a role in filopodia and lamelipodia structures. In addition, the presence of the kinase OSR1 (OXSR1) in association with PrM may further argue in favor of the notion that such a circuitry might be operational. OSR1 is a SOK1-related kinase that phosphorylates various chloride channels, PAK1 and RELT under conditions of environmental stress [38,39]. PAK1 is the upstream kinase controlling the effector function of CDC42 and Rac1 functioning in the dissolution of stress fibers and reorganization of focal complexes [40] and mutation of the putative residue in PAK1 phosphorylated by OSR1 reduced the activation of PAK1 by CDC42.

This interpretation, which could account for this scenario taking place in dendritic cells for example, does not exclude an alternative interpretation when infection is occurring in endothelial cells. In this case, not only viral entry would benefit from inhibition of Rho activation, but Rho inhibition could also contribute to the disorganization of stress fibers subjacent to the adherens junctions facilitating vascular leakage, as seen in dengue-infected endothelial cells [41]. In the same way, the PrM associating protein TSPAN3 and its cognate interactors Claudin11 (CLDN11) and integrin-beta1 (ITGB1) that are involved in the formation of tight junctions, structures suspected of being deregulated upon dengue infection [41] (see above), may also contribute to the hallmark vascular leakage in DHF.

Another biological process that seems to be targeted is the nuclear import/export module as evidenced by the association of Env with Importin13 (IPO13), a nuclear transport protein belonging to the importin-beta family. IPO13 is known to mediate the import of UBC9, PAX3, PAX6 (and others), as well as the nuclear export and cytoplasmic release of eIF-1A [42,43], a translation initiation factor that reportedly binds to the 3'SL of the DENV4 and West Nile virus genomic RNA [44,45]. One may suspect that as eIF-1A release in the cytoplasm is dependent upon binding of an import cargo to IPO13, binding of Env to IPO13 must stimulate eIF-1A release, which is presumed to serve as a bridge enhancing the association of the 3'and 5'UTR of the viral RNA so to facilitate cap- dependent and independent translation [45]. Although dengue virus NS5 and West Nile capsid proteins are normally imported into the nucleus [46,47] to date there are no reports of the Env protein being imported.

As virus entry, disassembly and packaging are tightly choreographed, fine tuned conditions are required to maximize viral cycling. One critical parameter is the regulation of the pH of virus containing organelles [48]. During viral entry the membrane fusion apparatus is set in gear by the acidic pH of the endosome [49] and, during provirus assembly in the ER and then the Golgi, pH is one major determinant of infectivity, as furin mediated cleavage of PrM is only possible around pH 5-6.0. Elevation of the pH at these steps renders the incoming virus infection-defective and the secreted virus infectivity is decreased by three orders of magnitude [49]. Thus tight control of the endosomal and secretory pathway pH is necessary for the virus to achieve maximal throughput. Vacuolar-type H+-Atpase (V-ATPase) is a major proton pump regulating pH homeostasis, which is essential for vesicular trafficking. The V-ATPase is a multimeric complex with several subunits some of which functions to recruit the remaining members of the complex in an organelle-specific fashion [50]. The V-ATPase generates an electrochemical proton gradient and in endosomes it promotes an eletrophoratic chloride transport via the CLC5 exchanger, to increase the acidity of the compartment [51]. In our assay, PrM interacted with the proposed intracellular chloride channel protein CLIC2, and CLIC2 associates with the Golgi tethering factor TRAPPC2. This protein is localized to Golgi buds and COPI-coated vesicles, and is part of the TRAPP complex, that specializes in vesicle and other intracellular cargo transport [52]. Thus it is possible that the PrM-CLIC2-TRAPPC2 interaction would be involved with the late virus maturation processes that take place in the late Golgi. Whether CLIC2 plays a role similar to CLC5 in the acidification of this particular set of organelles is an interesting possibility, and we plan to investigate it further as for another flavivirus, Murray Valley Encephalitis, virions with particle-bound prM were up to 400-fold more resistant to low pH environment, a fact that might have important consequences for E conformational changes in the acidic exocytic pathway [53]. Nevertheless such a mechanism has been proposed before for the proton-transporting, osteoclast ruffled membrane, where the CLIC2 homologue CLIC5B may be coupled to the ATPase to form the eletrogenic complex acidifying the bone reabsorption compartment [54].

Another aspect of PrM-host protein interaction is its association with RKIP (PEBP1). Initially identified as a RAF-1 kinase inhibitor, it is now known to be involved in the inhibition of several signaling pathways, including the MAPK, G-protein coupled receptor (GPCR) and NF-KB pathways [55,56]. The requirement for NFKB activation as a component of IFN-beta regulation is well accepted and is an essential part of the innate immune response against viral infection. Thus association of PrM with RKIP opens up the possibility of another mode of viral subversion of the innate cellular response to infection by modulating the activity of upstream kinases in the NFKB signaling pathway induced upon recognition of viral RNA by the TLR-RIG-I system. Several reports on global gene expression profiles in patients, as well as *in vitro *models of infection, report a strong bias toward the up-regulation of immune response, inflammation, metabolism and response to wounding ontology genes and down-regulation of cytoskeletal and curiously, NF-KB-related genes [57-59]. The immune-associated genes were mostly interferon-related as expected, given the strong association between RNA virus infection and the RLR-IFN or TLR3/7-IFN pathways. Nevertheless there were notable differences between samples from uncomplicated DF and severe DSS patients regarding immune-related genes as DF samples show a robust up regulation of IFN pathway related genes while DSS samples do not [59]. Curiously gene ontology analysis of our dengue structural proteins network does not show direct association with IFN pathway GO terms but it does with response to wounding and inflammation (**Table **3**and Figure **2B). Despite the lack of GO association with the immune response/IFN pathways, the association of PrM with the RKIP kinase is intriguing as this kinase associates with and inhibits the TAK-1/NIK NFKB kinases that usually operate downstream of the Toll receptors 3 and 7 (TLR3/7) and TNF receptors, mediating NFKB -dependent as well as IRF7-dependent gene activation [60,61]. Both TLR3 and TLR7 are used during dengue infection [62,63] and levels of TNF-alpha and TNFRI have been correlated with hemorrhagic manifestations [25]. As induction of IFN-alpha gene family is dependent on IRF7, it is formally possible that the PrM-RKIP (PEBP1) complex might be inhibiting TAK1/IKK-dependent IRF7 phosphorylation and activation, possibly downstream of the TLR3/7 receptors, negating the expression of IFN-alpha and therefore, IFN-alpha dependent genes. It may play a similar role downstream of the TNF receptor. In addition, there is a connection in our interaction network between two pathways that respond to RNA-dependent innate anti-viral response, the IKK-NFKB pathway, described above, and the JNK2-AP1 pathways. Here these two components are brought together via the concomitant association of MAPK1 with RKIP and TOB1, both PrM primary interactors. Chu *et al*. describes a condition where both these pathways are essential for efficient induction of type I IFN [64]. The possible subduction of the TLR pathway is not an exclusivity of the human host as the same pathway controls dengue virus infection in the *Aedes aegypti *vector along with a JAK-STAT pathway [65]. Interestingly, Reumer *et al*., recently demonstrated that the human RKIP ortholog, Drosophila PEBP1 is involved in a conserved immune defense pathway in the fly, downstream of the TLR receptor and possibly a JAK-STAT pathway [66]. Put together, the interaction of PrM with RKIP (PEBP1) seems to be involved in a conserved innate immune response pathway that may operate both, in the mosquito vector and in the human host, likely to the advantage of the virus. Probably related to the same pathway, we find an association between PrM and HMGXB3. This protein belongs to the high mobility group box proteins (HMGBs), which besides their well described functions in chromatin remodeling and transcription, have recently been linked to inflammation and the initiation of innate immune responses [67]. Yanai *et al*. recently demonstrated that HMGB1, 2 and 3 bind to many kinds of immunogenic nucleic acids recognized by TLR3, 7 and 9, and that HMGB1 and 2 knockout mouse cells are defective in interferon induction by TLR-targeted DNA and RNA [68]. In addition dengue infected macrophages secrete large amounts of HMGB1 [69].

We find that the Env protein of DENV2 associates with hemopexin (HPX) but the significance of this interaction remains to be deciphered. The hepatitis E virus structural component Orf-3 has also been shown to bind HPX and SIV and HCV infected subjects show an increased level of hemopexin (HPX) expression, which has been linked to disease [70-72]. One possible link to the pathology of DHF is that adult patients with dengue show high levels of oxidative stress, which also may be associated with vascular leakage [73]. Production of ROS by endothelial cells and immune cells have been reported to occur after dengue virus infection [73] and HCV infection leads to a four-fold decrease in heme-oxygenase (HO-1) in the liver and in cell lines stabling expressing the HCV core protein [74]. HO-1 mRNA is normally induced by receptor-mediated signaling after uptake of heme by macrophages, hepatocytes and endothelial cells, a function prevented by hemopexin. Hemopexin is considered an acute phase protein and is regularly induced by acute phase cytokines, IL-1 and TNF-alpha [75]. Thus binding of hemopexin to DENV2 ENV might disturb such a circuitry aggravating oxidative stress in the blood vessels endothelial lining. Interestingly, Xi *et al*. reported decreased expression of several heme-oxygenases in dengue-infected *Aedes aegypti *in a NF-KB dependent fashion [76].

As is the case with HIV and many other small RNA viruses, a compact genome demands maximal interaction with the diverse cell machineries in order to successfully produce a progeny. Such intense interactions may have unforeseen consequences depending on viral loading, host genetic background and other relevant factors [77] and may create havoc at the system level and deeply disturbing the operation of a cell and eventually tissues and organs. It is well accepted that changes in protein abundance and post-translational modifications can function as switches in the interactome and a viral infection of a cell can fulfill, perhaps more than any other condition, the requirements for the dynamic perturbation of the cell's interaction network, a situation believed to be central in the development of disease. Thus, depending on variables such as initial viral load and rate of viral replication, the abundance of viral proteins interacting with the preexisting network may change dramatically and significant increases in a protein abundance have been known to cause a increase in its interaction promiscuity [78] whose biological effects will vary depending on the role of the host interaction partner in the network, whether they represent hubs or non-hubs. We analyzed several microarray experiments deposited in the GEO database for dengue infections both in vitro and in vivo (e.g., GSE18090, GSE9378 and GSE13052). To our surprise only 8 out of the 31 proteins obtained in the two-hybrid assay, RUFY3, MBP, TMEM167B, TSPAN3, OXSR1, TOB1, CLU and HLA-B were differentially expressed in at least one of the microarray studies. Only CLU and HLA-B were up regulated upon infection and all the others were down regulated, suggesting that the virus structural proteins tend to associate with proteins coded by genes whose expression is stable under infection conditions. When we expanded our search for the genes at the level 2 of the D2-human interactome, we found that at least 50% of the genes coding for the proteins associated with TOB1, OXSR1, RUFY3, HLA-B, MBP, TSPAN3, MPRIP, NRGN and PEBP1 were differentially regulated upon infection. On the other hand less than 20% and, in many instances, none of the genes for the proteins associated with F8, PLG, ALDOB, TMOD1 and others were regulated. Remarkably more than 85% of all the regulated genes related to the D2-human interactome were down-regulated upon infection (results not shown).

## Conclusions

In summary, we present data that support a scenario where DENV2 structural proteins interact with diverse cellular proteins representing many biological functions, but with its greatest impact pointing toward the interference with coagulation and inflammatory pathways. The conditions by which dengue proteins interact with these pathways are manifold and range from direct interactions with effector cascades as well as with structural components serving as targets for those cascades. Although the use of analytical tools to decode protein-protein interaction networks is of great help for the generation of hypothesis and the prevision of biologically relevant scenarios, our inability to incorporate into the network, biological variables such as time of expression, concentration and affinity of network components precludes us from getting a more accurate view of the inner workings of a dengue infection upon the host's biology. Nevertheless the approximation allowed by this relatively new methodology gives us previously unattainable views of the global behavior of a host's biological network during a loss of homeostasis enforced by the sudden introduction of alien proteins resulting from a virus infection. The observation of these very changes in connectivity may shed new light on the etiology of broad pathogenesis traits seen in infected individuals and stir up the search for new therapeutic strategies.

## List of abbreviations

Cap: Capsid; Env: Envelope; PrM: Pre-membrane; D2-H: Dengue 2 human interactions; DENV2: Dengue virus type 2.

## Competing interests

The authors declare that they have no competing interests.

## Authors' contributions

BBF carried out the two-hybrid screens and interpretation of the data; AMWS help design the experiments and performed statistical analysis; CGF helped draft the manuscript and gave fundamental intellectual contributions; LRBS designed the experiments, analyzed the data and wrote the manuscript.

## Pre-publication history

The pre-publication history for this paper can be accessed here:

http://www.biomedcentral.com/1471-2334/11/34/prepub

## Supplementary Material

Additional file 1 **Supplemental Figure S1 - Schematic representation of the complement and coagulation pathway adapted from Wikipathways and possible sites of interference from the Dengue structural proteins as discussed in the text**. Red arrows and (+) signs suggest an additive effect and T-bars a negative effect. Click here for file
