# Supplementary figures and images for: Dengue-2 Structural Proteins Associate with Human Proteins to Produce a Coagulation and Innate Immune Response Biased Interactome
Source: BMC Infect Dis. 2011 Jan 31;11:34. doi: 10.1186/1471-2334-11-34 (PMC3037883; doi:10.1186/1471-2334-11-34)

## Slide 1
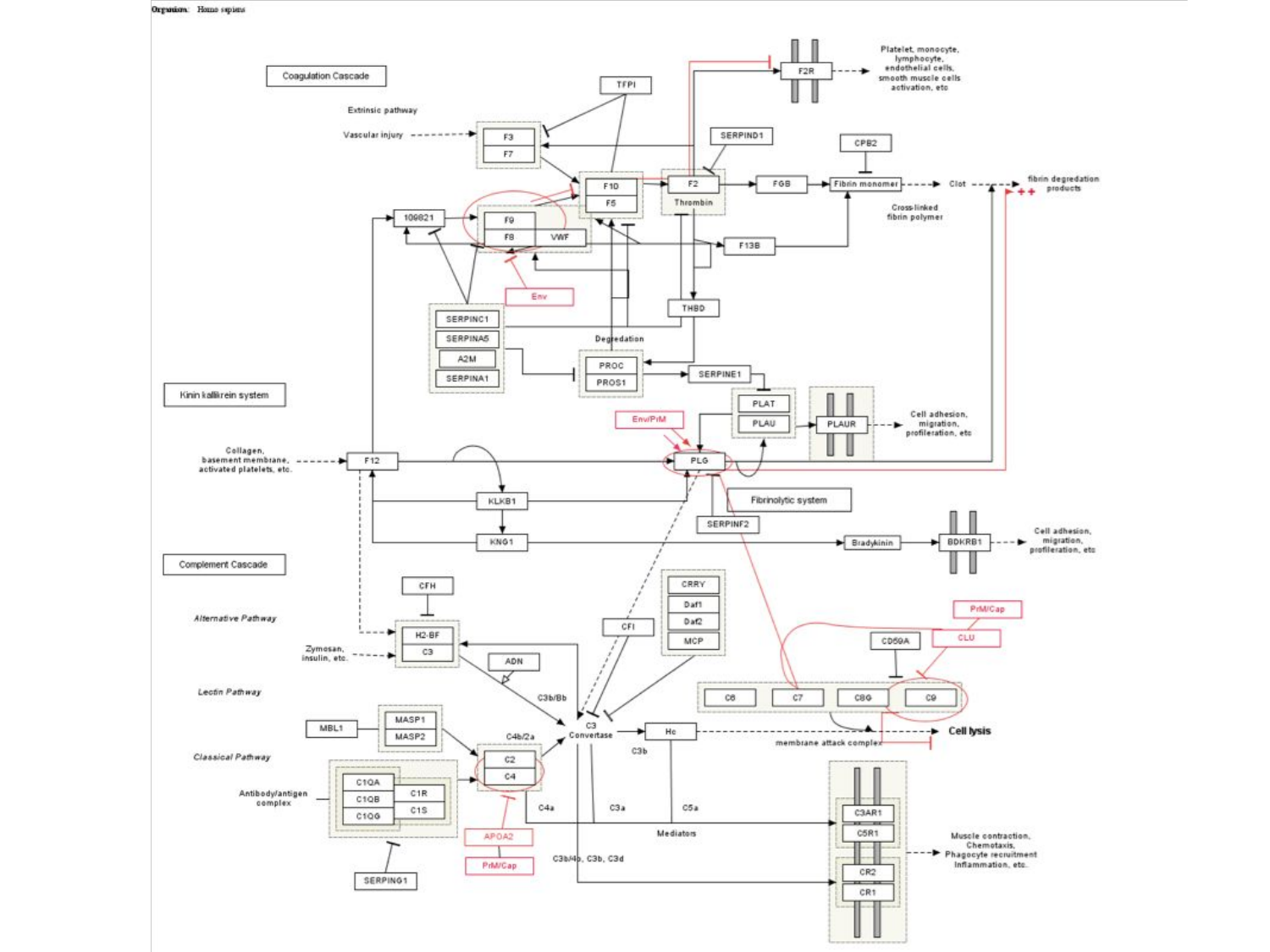

Supplement: Additional file 1 — Supplemental Figure S1 - Schematic representation of the complement and coagulation pathway adapted from Wikipathways and possible sites of interference from the Dengue structural proteins as discussed in the text. Red arrows and (+) signs suggest an additive effect and T-bars a negative effect. [file 1471-2334-11-34-S1.PPT]
